# Supplementary material for: Evaluation of Soybean Wildfire Prediction via Hyperspectral Imaging
Source: Plants (Basel). 2023 Feb 16;12(4):901. doi: 10.3390/plants12040901 (PMC9967622; doi:10.3390/plants12040901)
Supplement: Supplementary file 1 [file plants-12-00901-s001.zip › plants-2164125-supplementary.pdf]

Table S1. Formula table for classification equation accuracy verification

**Cheongja 3-ho**		**Daechan**	
Selected Function	Accuracy (%)	Selected Function	Accuracy (%)
1	81.90	1	65.87
2	61.21	2	68.75
**3**	**95.69**	**3**	**100**
4	81.90	4	65.87
5	80.17	5	70.19
6	81.90	6	75.00
7	80.17	7	70.19
8	79.31	8	67.79
9	80.17	9	72.60

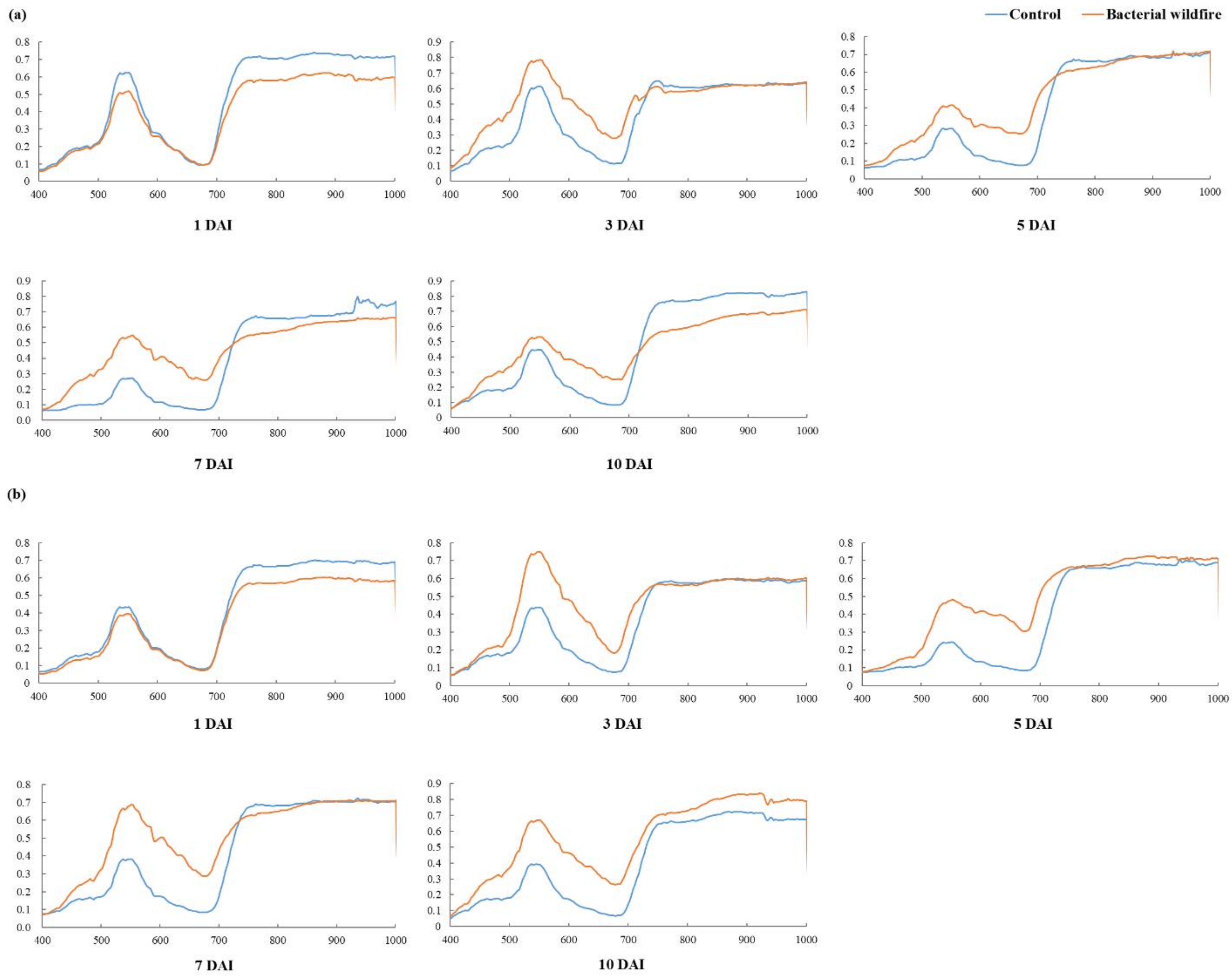

Figure S1. Spectral reflectance of healthy and diseased soybean leaves of the (a) Cheongja 3-ho and (b) Daechan varieties.
